# Muscle Bioenergetics and Cognitive Executive Function: The SOMMA Study Baseline

**DOI:** 10.1093/geroni/igab046.483

**Published:** 2021-12-17

**Authors:** Stephen Krtichevsky, Steve Cummings, Anne Newman, Paul Coen, Anthony Molina, Russell Hepple, Peggy Cawthon

**Affiliations:** 1 Wake Forest School of Medicine, Wake Forest School of Medicine, North Carolina, United States; 2 Wake Forest School of Medicine, Winston Salem, North Carolina, United States; 3 California Pacific Medical Center, San Francisco, California, United States; 4 University of Pittsburgh, Pittsburgh, Pennsylvania, United States; 5 AdventHealth, Orlando, Florida, United States; 6 UCSD, La Jolla, California, United States; 7 University of Florida, Gainsville, Florida, United States

## Abstract

Better executive function has been associated with faster walking speed, but the basis for this association is unclear. Systemic factors appear to contribute mitochondrial function across multiple tissues including muscle and brain. We hypothesized that muscle-based measures of bioenergetics capacity would be associated with cognitive function at SOMMA’s baseline. MRI-based ATPMAX and muscle fiber respirometry-based max OXPHOS were correlated with scores on the MoCA (mean: 24.0; SD: 3.2); Trails B (mean: 138 seconds; SD: 73) and the Digit Symbol Coding Tests (mean: 50.8; SD: 14.9). The spearman correlations between ATPmax and the three measures were: 0.10 (p=0.29); -0.20 (p=0.03) and 0.16 (p=0.09), respectively. The association between max OXPHOS were: 0.18 (p=0.02); -0.20 (p<0.01) and 0.11 (p=0.13), respectively. Some associations appeared stronger in men than women. Gender interactions and whether energetics mediate some of the association between cognitive function and gait speed will be explored in the full baseline sample.

